# Immunohistochemical Expression of the Stem Cell Marker CD133 in Colorectal Carcinoma

**DOI:** 10.7759/cureus.41242

**Published:** 2023-07-01

**Authors:** Sweta Suman, Subhransu Kumar Hota, Pranati Misra, Nageswar Sahu, Subrat Sahu

**Affiliations:** 1 Pathology, Kalinga Institute of Medical Sciences, Bhubaneswar, Bhubaneswar, IND; 2 Pathology, Hi-Tech Medical College and Hospital, Bhubaneswar, Bhubaneswar, IND; 3 Surgery, Kalinga Institute of Medical Sciences, Bhubaneswar, Bhubaneswar, IND

**Keywords:** pathological parameters, cd133, immunohistochemistry, cancer stem cell, colorectal carcinoma

## Abstract

Background

Colorectal carcinoma (CRC) is the second-leading cause of cancer-related death. Despite the combined (surgery, chemotherapy, radiotherapy, and immunotherapy) modalities of treatment, the prognosis remains poor, mostly because of recurrence and distant metastasis. Cancer stem cells (CSC) are thought to be responsible for the development and spread of tumors. Hence, targeted therapy against these cells hopes to reduce the chance of recurrence and metastasis and improve the prognosis. Many immune markers have been identified to detect CSC in CRC. Here, we tried to assess the immunohistochemical expression of the stem cell marker CD133 in colorectal carcinoma and its correlation with various pathological parameters.

Methodology

A total of 51 cases of CRC were analyzed. Immunohistochemistry for CD133 was done after standardization in our laboratory. Expression status was decided based on the total score obtained by multiplying the intensity score by the percentage score. CD133 expression was correlated with the age and gender of the patient, tumor location, histological grade, extent of invasion, lymphovascular invasion (LVI), perineural invasion (PNI), and nodal status.

Results

High CD133 expression was seen in 21 (41.17%) cases. There was no significant association between CD133 expression and the pathological parameters except the tumor site. CD133 expression was significantly higher as we moved from the proximal colon to the rectum.

Conclusions

CD133 expression was significantly higher in the distal part of the large intestine as compared to the proximal part. But there was no linear correlation between CD133 expression and histological grade, extent of invasion, or nodal status.

## Introduction

Colorectal carcinoma (CRC) is the most common malignancy of the gastrointestinal tract [1]. It is the third most prevalent cancer and ranks second in cancer death rates in the world. As per GLOBOCAN 2020 data, the estimated CRC disease burden in 2020 will be 1.9 million new cases and 0.9 million deaths [1]. The incidence of CRC in India is gradually increasing, causing a national health burden. From 2004 to 2014, the incidence of colorectal carcinoma in India increased by 20%. One of the important risk factors is adaptation to the Western lifestyle [2,3]. Nearly 70% of CRCs are sporadic, with no inherited genomic abnormalities [4]. About 80-85% of CRCs are due to chromosomal instability [5]. The high mortality in this malignancy is commonly due to distant metastasis, local recurrence, and failure to respond to chemotherapy [6].

Cancer stem cells (CSC) were first demonstrated in cases of acute myeloid leukemia (AML). Recent studies show that cancer stem cells are a subset of stem cells responsible for tumorigenesis, metastasis, and relapse [7]. At present, surgery is the mainstay of therapy for CRC, which spares the cancer stem cells, leading to an increased risk of recurrence. Targeted therapy against cancer stem cells may halt tumorigenesis and even eradicate the tumor. Identification of cancer stem cells is a current topic of interest for their potential use in the treatment of CRC [8,9]. The finding of cancer stem cells is extremely difficult in routine hematoxylin and eosin (H&E)-stained tissue sections. A newer method like immunohistochemistry is helpful for the identification of these CSCs. Many CSC markers are the topic of research nowadays. Among all these markers, CD133, OCT-4, SOX-2, and NANOG are the ones commonly analyzed in colorectal carcinoma [10,11].

CD133, also known as Prominin-1, is a pentaspan transmembrane glycoprotein whose expression was first found in hematopoietic stem cells. Immunohistochemical detection of CD133-positive cells has been used for the isolation of CSC in both hematopoietic and non-hematopoietic tumors [12,13]. In colorectal carcinoma, CD133 is located in the luminal content, apical surface, and cytoplasm. Multiple pathways like WNT, TGF-β, Notch, and Hedgehog signaling are found to be associated with CSCs and CD133 expression in colorectal cancer. The WNT pathway plays an important role in the growth and maintenance of CSCs. It also plays an important role in CRC development. It has been found that this pathway is activated in CD133-positive cells. The TGF-β pathway has a tumor suppressor effect in healthy tissues but acts as a promoter in colorectal cancers. Notch signaling plays a role in the differentiation and self-renewal of CSCs. Hedgehog signaling, which is active in CD133-positive cancer stem cells, promotes colon cancer growth, stem cell self-renewal, and metastatic behavior in advanced cancers. CD133 plays a role in cell-cell and cell-matrix interactions, facilitating tumor invasiveness. CD133 is widely used as a marker to identify and isolate colorectal cancer stem cells. It has also been investigated for a better understanding of the characteristics and functions of cancer stem cells in CRC [14]. Recent colon cancer studies suggest CD133-expressed cells have a role in invasiveness and differentiation capacity [13]. Hence, we have analyzed the expression of the CSC marker CD133 in colorectal carcinoma and its correlation with different pathological parameters, including tumor extension and differentiation (histological grade).

## Materials and methods

All the histologically confirmed cases of primary CRC from April 2021 to September 2022 were included in this study. Recurrent cases and cases with neoadjuvant therapy or poorly preserved samples were excluded. In each case, the following clinicopathological parameters were noted: age, gender, location (site) of the tumor, histopathological type, histopathological grade, microscopic tumor extension, lymphovascular invasion (LVI), perineural invasion (PNI), and lymph node metastasis. The study was done after approval from the Institutional Ethics Committee (KIIT/KIMS/IEC/494/2020).

Immunohistochemical evaluation of CD133 was done by the secondary labeling method on formalin-fixed paraffin-embedded tissue sections (4-5 µm thick) on poly-L-lysine coated slides. Deparaffinization was done by placing the slides on the hot plate at a temperature of 60 degrees for one hour. For the antigen retrieval, slides were treated by microwave heating in citrate buffer (pH 6.0) for 20 minutes of incubation with 0.3% hydrogen peroxidase. Slides were treated with rabbit polyclonal CD133 antibody (dilution 1:400, 18470-1-AP, Proteintech). The streptavidin-biotin-peroxidase kit was used to treat sections, and diamino-benzidine (DAB) was used to find the reaction product after incubation. Finally, the sections were counterstained with hematoxylin and mounted. A negative control was prepared on one of the test blocks by omitting the step of primary antibody. Kidney tissue was taken as the positive control.

**Figure 1:**
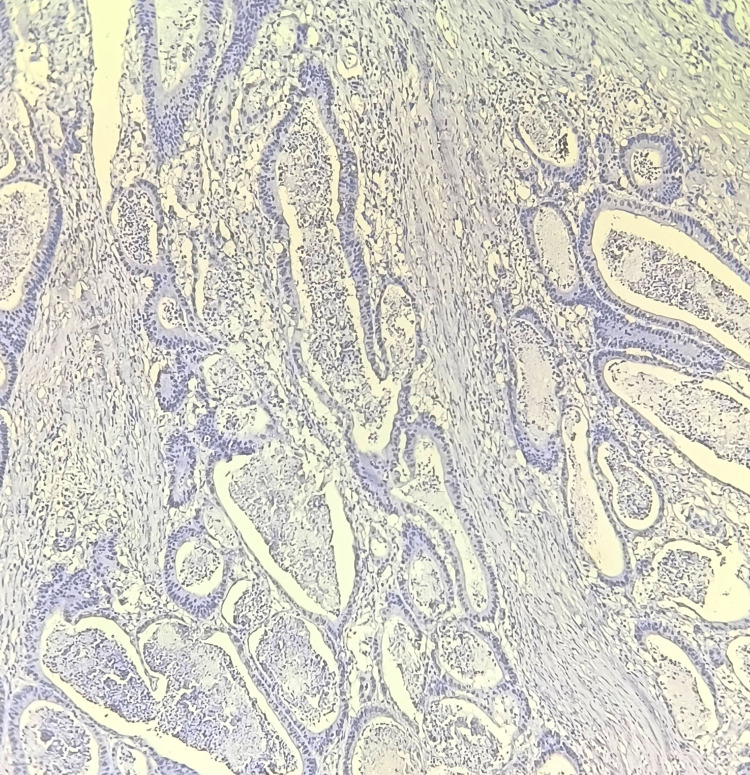
Immunohistochemistry for CD133: no staining Total score = 0

**Figure 2:**
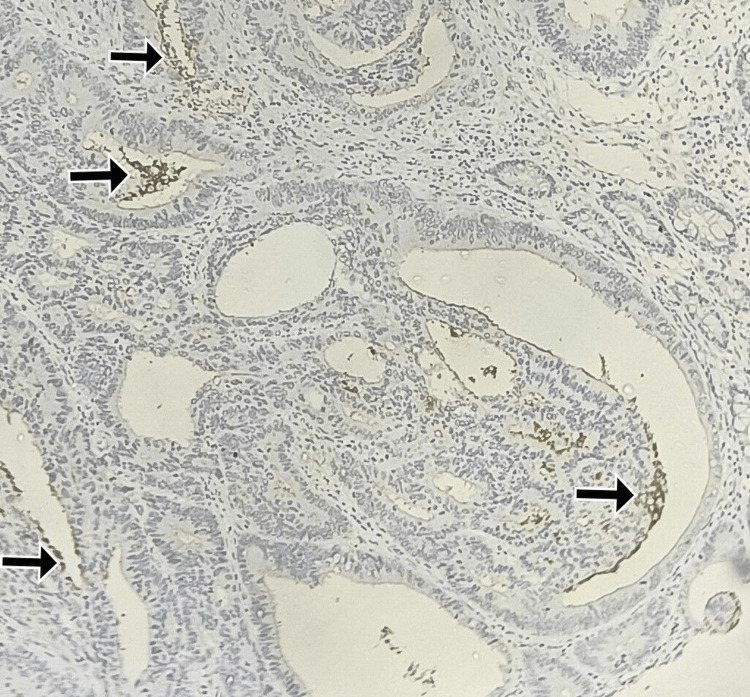
Immunohistochemistry for CD133: moderate luminal and intraglandular debris staining in 10-20% of cells Total score = 2

**Figure 3:**
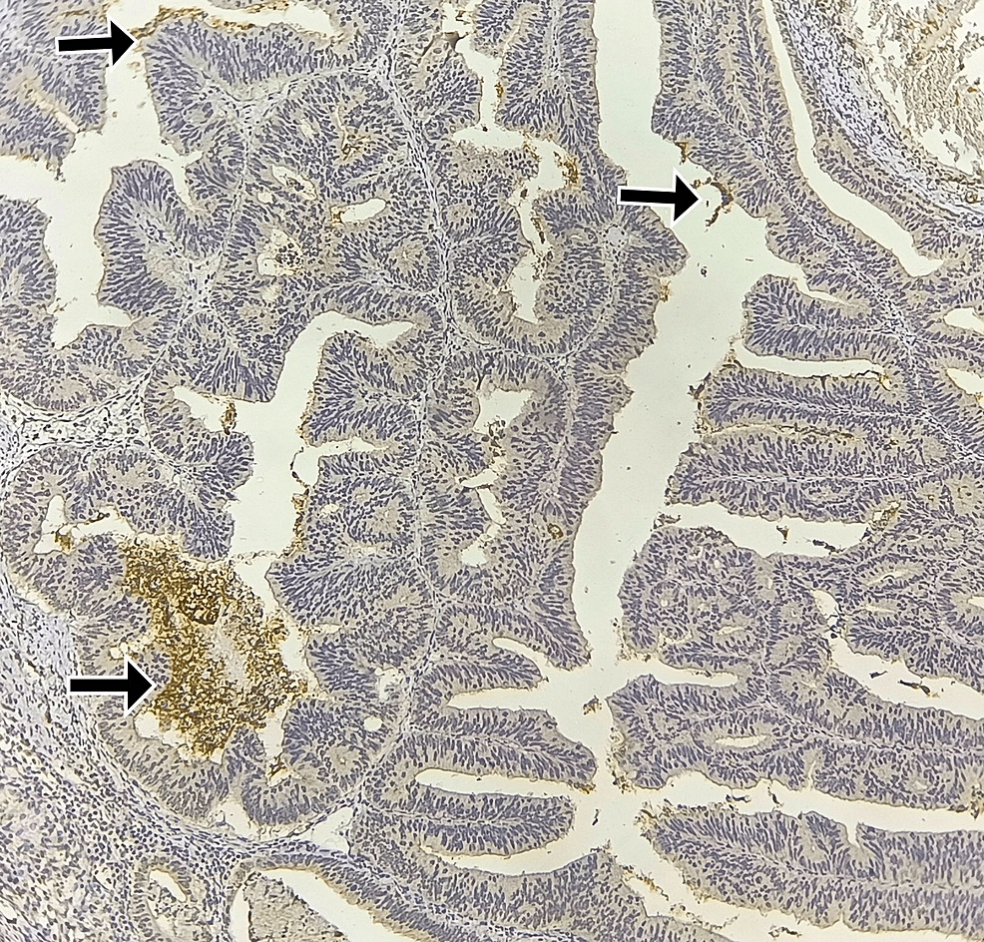
Immunohistochemistry for CD133: strong luminal and intraglandular debris staining in 10-20% of cells Total score = 3

**Figure 4:**
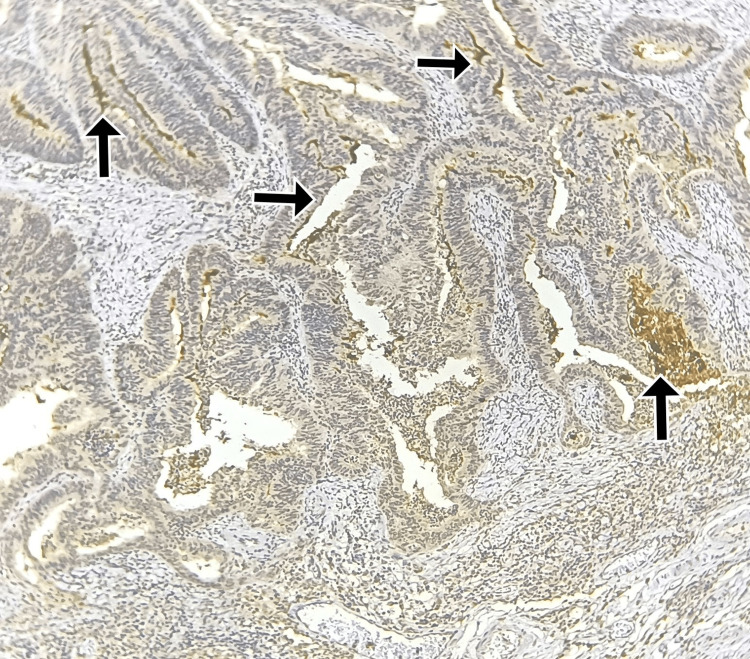
Immunohistochemistry for CD133: Strong luminal and intraglandular debris staining in >50% of cells Total score = 9

For CD133 expression analysis, cytoplasmic membranous or luminal and intraglandular debris staining was taken as positive staining. For quantitative analysis, the percentage of CD133-positive cells was scored as 0: <10%, 1: 10-20%, 2: 21-50%, and 3: >50%. For qualitative analysis, CD133 immunoreactive staining intensity was scored as 0: negative, 1: weak staining, 2: moderate staining, and 3: strong staining. A total score of 0-9 was obtained by multiplying the percentage of stained cells by the staining intensity. A total score of 0 was taken as negative, 1 and 2 as weak, 3 to 6 as moderate, and 7 to 9 as strongly positive [15]. Negative staining and weak positives (total score ≤2) were taken as low expressions (Figures 1-2). Moderate and strong positive cases (total score ≥3) were taken as high expressions (Figures 3-4).

Statistical analysis

Data were entered into a Microsoft Excel spreadsheet in 2016 (Microsoft® Corp., Redmond, WA) and analyzed using EpiInfo software version 7.1.3. Results were expressed as mean, median, standard deviation, percentage, frequency, and proportion. The chi-square test or Fisher's exact test were used as tests of association. A p-value of <0.05 was taken as statistically significant.

## Results

A total of 51 cases were analyzed in this study. The clinicopathological data are listed in Table 1. The patients were aged between 29 and 85 years, with a mean age of 58.67 (±13.84) years. The study population was comprised of 39 (76.5%) males and 12 (23.5%) females. The most common site was the right colon (47.1% of cases), followed by the left colon (41.2% of cases), and the rectum (11.8% of cases).

**Table 1:** Clinicopathological parameters of all patients T: tumor extension, PNI: perineural invasion, LVI: lymphovascular invasion, N: nodal metastasis

Parameters	Number (percentage)
Age (years)	
<58	27 (53.0)
>58	24 (47.0)
Gender	
Male	39 (76.0)
Female	12 (24.0)
Tumor site	
Right colon	24 (47.0)
Left colon	21 (41.0)
Rectum	06 (12.0)
Histological grade	
Grade I	25 (49.0)
Grade II	21 (41.0)
Grade III	05 (10.0)
T	
T1	04 (08.0)
T2	05 (10.0)
T3	40 (78.0)
T4	02 (04.0)
N	
N0	25 (49.0)
N1	15 (29.0)
N2	11 (22.0)
N3	00 (00.0)
LVI	
Present	15 (29.0)
Absent	36 (71.0)
PNI	
Present	11 (22.0)
Absent	40 (78.0)

High CD133 expression was detected in 21 (41.17%) cases, while low expression was seen in 30 (59%) cases. The association between CD133 expression and different parameters is depicted in Table 2. The incidence of high CD133 expression was highest among the tumors located in the rectum (83.3%), followed by the left colon (47.6%) and right colon (25.0%), showing a significantly higher CD133 expression in tumors located in the distal part. No linear trend was observed between CD133 expression and T stage, but the association between these two parameters was significant (p=0.041). The chance of high CD133 expression was highest in the T4 stage (100%), followed by T1 (75%), and T3 16(40%). No significant correlation was observed between CD133 expression and other clinicopathological parameters like age, gender, histological grade, LVI, PNI, and nodal metastasis.

**Table 2:** Association between CD133 expression and different clinicopathological parameters T: tumor extent, N: nodal metastasis, LVI: lymphovascular invasion, PNI: perineural invasion

Parameters (n)		CD133 expression		p-value
		High number (%)	Low number (%)	
Age	<58 years (n=27)	11 (40.7)	16 (59.3)	0.947
	>58 years (n=24)	10 (41.7)	14 (58.3)	
Gender	Male (n=39)	18 (46.2)	21 (53.8)	0.193
	Female (n=12)	03 (25.0)	09 (75)	
Tumor site	Right colon (n=24)	06 (25.0)	18 (75.0)	0.025
	Left colon (n=21)	10 (47.6)	11 (52.4)	
	Rectum (n=06)	05 (83.3)	01 (16.7)	
Histological grade	Grade I (n=25)	10 (40.0)	15 (60.0)	0.522
	Grade II (n=21)	10 (47.6)	11 (52.4)	
	Grade III (n=05)	01 (20.0)	04 (80.0)	
T stage	T1 (n=04)	03 (75.0)	01 (25.0)	0.041
	T2 (n=05)	00 (00.0)	05 (100.0)	
	T3 (n=40)	16 (40.0)	24 (60.0)	
	T4 (n=02)	02 (100.0)	00 (00.0)	
N stage	N0 (n=25)	11 (44.0)	14 (56.0)	0.333
	N1 (n=15)	04 (26.7)	11 (73.3)	
	N2 (n=11)	06 (54.5)	05 (45.5)	
LVI	Present (n=15)	08 (53.3)	07 (46.7)	0.255
	Absent (n=36)	13 (36.1)	23 (63.9)	
PNI	Present (n=11)	03 (27.3)	08 (72.7)	0.290
	Absent (n=40)	18 (45.0)	22 (55.0)	

## Discussion

As per GLOBOCAN 2020 data, CRC is the third most prevalent (10% of all cancers) malignancy worldwide. Also, it is the second-leading cause of cancer mortality, accounting for 9.4% of all cancer deaths. The disease burden of CRC in India is also high, accounting for 1.3 million new cases and 0.8 million deaths in the year 2020 [1]. The incidence of CRC has been increasing, which may be due to environmental changes and westernization [2,3]. Surgical resection is the mainstay of treatment. Neoadjuvant therapies like chemotherapy, radiotherapy, and immunotherapy have also been tried to reduce the tumor mass, but there is always a risk of recurrence [16]. Despite these therapeutic modalities, the prognosis is bad, mostly because of relapse and metastasis, which are seen in nearly 50% of cases [17]. So to eradicate these cancer cells, studies are going on to identify cancer stem cells and develop newer therapeutic modalities.

Newer evidence suggests that CSCs, also called tumor-initiating cells (TICs), are a small subset of cells responsible for tumorigenesis, metastasis, and relapse [18]. Colonic stem cells are located at the crypt base among other cells. Colon CSCs develop due to the accumulation of genetic and epigenetic alterations in progenitor cells or due to the dedifferentiation of somatic cells caused by genetic and environmental alteration. Colonic CSCs also have plasticity and heterogeneity properties, which are responsible for their self-renewal, differentiation, tumor progression, metastases, and drug resistance [16].

Multiple CSC immunomarkers have recently been used for the identification of CSC. In CRC, cancer stem cell surface markers and dysregulated or upregulated pathways can be used to target colon CSC for effective treatment [17].

CD133 is a transmembrane glycoprotein. Currently, many studies are going on using CD133 for the identification and isolation of CSCs in both hematopoietic and non-hematopoietic tumors. CD133-positive cells have the property of tumorigenesis along with self-renewal and differentiation capacity [13].

In this study, high CD133 expression was seen in 41.17% (21 out of 51) cases. We did not find a significant correlation between CD133 expression and age, gender, histological grade, LVI, PNI, or nodal metastasis. As we moved from the proximal to the distal colon, there was a significant (p=0.025) increase in CD133 expression (Table 2). The incidence of high CD133 expression was maximum in the rectum (83.3%) and lowest in the right colon (25.0%). Previous reports on CD133 expression in CRC at different sites show varied results. Similar to our results, Devrim et al. and Hong et al. also found significantly higher CD133 expression in tumors occurring in the distal part (rectum or left colon) [19,20]. Pop et al. and Park et al. also found increased CD133 expression in the distal part, but the difference was not statistically significant [21,22]. In contrast, Kostovski et al. and Ehteram et al. found higher CD133 expression in tumors occurring in the proximal part of the colon, but the difference was not significant [23,24]. However, none of these studies have explained the difference in CD133 expression at different sites of the large intestine.

We did not find a linear association between CD133 expression and the T-stage of the tumor. Though the expression was highest in the T4 stage, the T3 group showed lower expression than the T1 group. Hence, despite a significant p-value, no conclusive opinion can be made regarding the association between tumor extension and CD133 expression. This may be due to the uneven distribution of cases in different categories. The literature shows varied results regarding the association between CD133 expression and tumor extension. Kostavski et al. also found the highest CD133 expression in the T4 category with a significant p-value. But the association was not linear. CD133 expression was lower in the T2 group than in the T1 group [23]. However, Choi et al. found a linear increase in CD133 expression from the T1 to T4 groups with a significant p-value (p=0.024) [25]. In contrast, Hong et al. found significantly higher CD133 expression in T1 and T2 groups as compared to T3 and T4 groups [20].

## Conclusions

We did not find a significant association between CD133 expression and any of the pathological parameters except the tumor site. Expression was significantly higher in the distal part of the large intestine as compared to the proximal part. There was no linear correlation between CD133 expression and histological grade, the extent of invasion (T stage), or nodal status (N stage). These results fail to explain the significant role of stem cells in the pathogenesis of colorectal carcinoma. Hence, we think CD133 alone may not be adequate to detect CSCs and understand their role in the pathogenesis and prognosis of CRC. Further immunohistochemical studies of different CSC immune markers and comparison of the results with clinicopathological parameters are necessary to establish a novel panel of antibodies to detect CSC and predict tumorigenesis, malignant potential, and prognosis. It may also help plan the management of colorectal carcinoma.
